# Association Between Systemic Immunity‐Inflammation Index (SII) and Fatigue, Cancer, and Cancer‐Related Fatigue: Insights From NHANES (2005–2018)

**DOI:** 10.1002/cam4.70777

**Published:** 2025-03-17

**Authors:** Li Sun, Yanling Wu, Lydia Idowu Akinyemi, Zhiqiu Cao, Zhanhong Fan, Huahua Liu, Ziyi Yang, Leilei Zhang, Feng Zhang

**Affiliations:** ^1^ School of Nursing and Rehabilitation, Nantong University Nantong Jiangsu China; ^2^ Nantong Maternal and Child Health Care Hospital Nantong Jiangsu China; ^3^ Nantong Stomatological Hospital affiliated to Nantong University Nantong Jiangsu China

**Keywords:** Cancer, cancer‐related fatigue (CRF), Fatigue, NHANES, systemic immunity‐inflammation index (SII)

## Abstract

**Objective:**

To investigate the association between the systemic immunity‐inflammation index (SII) and fatigue, cancer, and cancer‐related fatigue (CRF) populations.

**Methods:**

The National Health and Nutrition Examination Survey (NHANES) from 2005 to 2018 provided data for this retrospective cross‐sectional study. By dividing the platelet count by the neutrophil count and the lymphocyte count, SII was calculated. Participants were categorized into four groups: normal, fatigue, cancer, and cancer‐related fatigue (CRF), with the normal group serving as the reference. Binary logistic regression was applied to assess the correlations. The dose–response relationship between SII and outcomes in the four groups was evaluated using restricted cubic splines. Use threshold effect analysis to determine the optimal SII value for each of the three groups. Stratified and subgroup analyses were performed based on sociodemographic factors and confounders, with specific attention to fatigue severity levels (mild, moderate, severe) in the fatigue and CRF groups.

**Results:**

Data analysis included a total of 32,491 participants, including 14,846 in the normal group, 14,581 in the fatigue group, 1520 in the cancer group, and 1544 in the CRF group. The results of binary logistic regression showed that SII was positively correlated with the fatigue group (1.43[1.33, 1.55]), cancer group (1.67 [1.43, 1.95]) and CRF group (1.93 [1.66, 2.25]). Restricted cubic spline analysis revealed a linear relationship between SII and outcomes. The threshold values (k) for each of these groups were identified as 464.78 × 10^3^ cells/μL, 448.97 × 10^3^ cells/μL, and 454.65 × 10^3^ cells/μL, respectively. Stratified analysis indicates that most groups exhibit significant differences. The subgroup analysis indicated that fatigue severity increased with higher SII levels, with the CRF group exhibiting the highest rate of severe fatigue (171% increase).

**Conclusion:**

SII is positively correlated with fatigue, cancer, and CRF in a linear way. Higher SII values are associated with greater fatigue, particularly in the CRF population.

## 1 Introduction

Cancer remains a major global public‐health challenge. In 2020, it claimed about 600,000 lives in the United States alone [1]. Chronic inflammation is a well‐known cancer hallmark [2], with 15%–20% of cancer cases are due to tissue damage from early‐stage infections, inflammation, or autoimmune responses. It involves complex interactions among cancer cells, stromal cells, and inflammatory cells, forming the “inflammatory tumor microenvironment” (TME) [3]. Interleukin‐6 (IL‐6) links inflammation to cancer and acts as a key information‐transmission hub [2]. Furthermore, a review highlights that tumor necrosis factor‐alpha (TNF‐α) strongly promotes cancer progression and metastasis [4], highlighting the role of inflammatory cytokines in cancer.

Increasing evidence links pro‐inflammatory states to fatigue [5]. Inflammatory mediators affect sleep [6], cognitive function [7], and mental health [8], harming social interactions [9] and daily activities [10]. The pathophysiology of fatigue is associated with inflammatory cytokines like serum C‐reactive protein (CRP) and interleukin‐1β (IL‐1β) [11]. In some autoimmune diseases, high peripheral inflammatory molecules can cross the blood–brain barrier and change neural transmission pathways [12].

Cancer‐related fatigue (CRF) is a major side‐effects of cancer treatment (CRF) [13]. Many patients remain exhausted after intensive therapy [14]. The National Comprehensive Cancer Network (NCCN) describes CRF as [15]: “An extreme sense of weariness caused by cancer or its treatment, which is persistent, not relieved by rest, and significantly impairs one's ability to function, often with variable activity levels.” CRF severely reduces the quality of life of patients and caregivers [16, 17], affecting physical health, psychosocial status, and economic or professional aspects [18, 19, 20, 21]. Numerous studies have found a link between CRF, inflammation, and immune responses [22, 23, 24]. IL‐6 and TNF‐α are crucial in CRF development. A large‐scale study on cancer survivors showed that systemic inflammatory markers (e.g., IL‐1α, IL‐1β, IL‐2, IL‐4, IL‐6, IL‐8, IL‐10, TNF‐α, and C‐reactive protein) are closely related to physical, emotional, and cognitive CRF [25]. These cytokines influence cancer progression and fatigue severity through central‐nervous‐system and neuroimmune mechanisms [26, 27]. Some studies target these cytokines with biological therapies to relieve CRF [28], focusing on reducing the pro‐inflammatory state or inhibiting key inflammatory signaling pathways [29, 30].

The systemic immune‐inflammation index (SII), first proposed by Hu et al. in 2014 [31], has been extensively explored and applied across various research fields since its inception. The index innovatively integrates three key inflammatory cell components—platelets, neutrophils, and lymphocytes—and quantifies them using the formula: divided by lymphocyte count, then multiplied by neutrophil count [32]. Compared to other methods for detecting cellular inflammatory factors, SII can be measured via a simple and fast routine blood test for early‐stage physiological‐state changes. It reflects systemic inflammation and local immunological responses ([33]). SII is associated with conditions like rheumatoid arthritis [34], hepatic steatosis [35], abdominal aortic calcification [36], and diabetes [37], and has prognostic value in cancer [38, 39].

We aim to explore the relationship between SII, fatigue, cancer, and CRF in NHANES, hypothesizing that higher SII values are associated with a greater incidence of these three groups.

## 2 Materials and Methods

### 2.1 Study Participants

**FIGURE 1:**
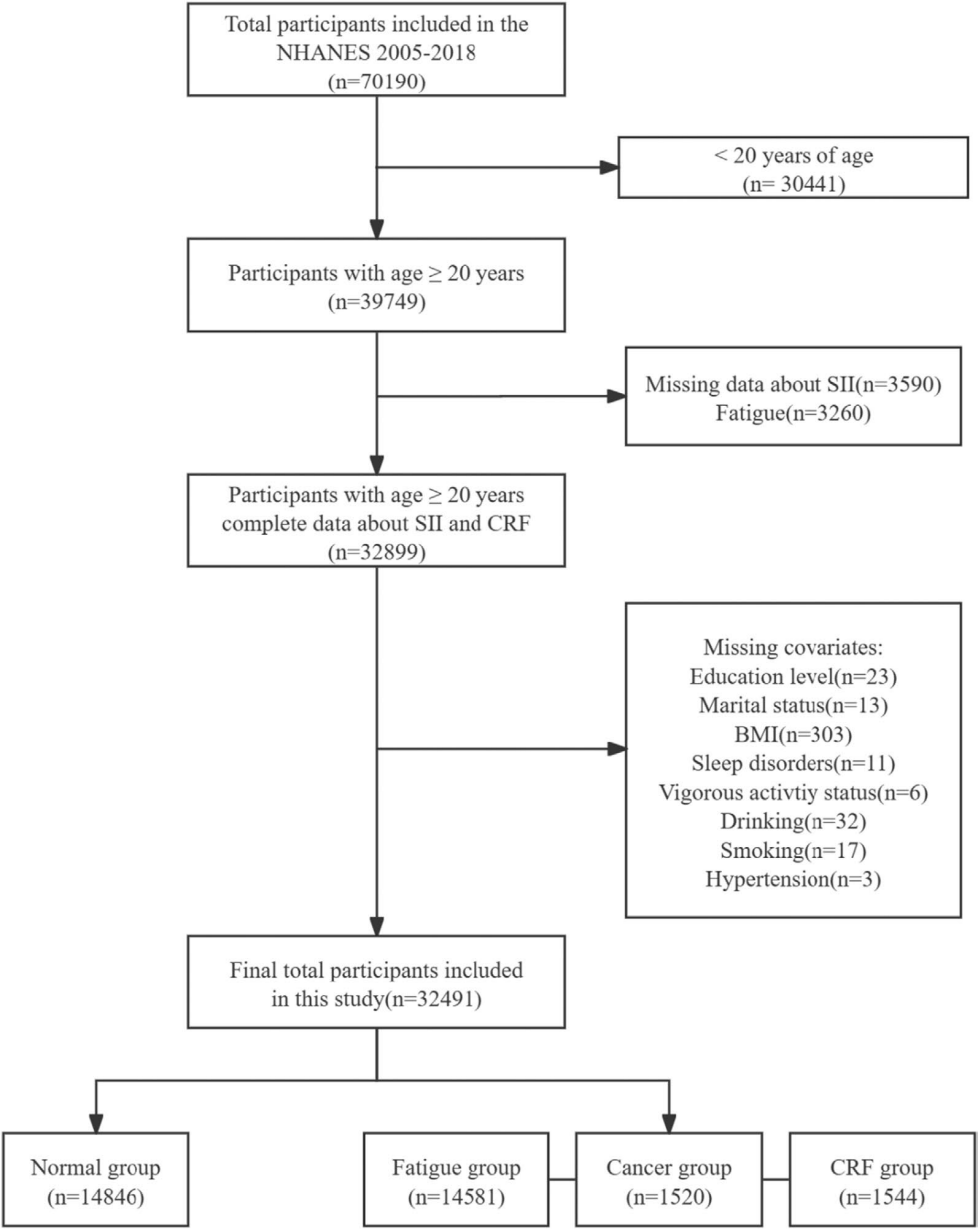
Screening flow chart of the research population. BMI, body mass index; CRF, cancer‐related fatigue; NHANES, National Health and Nutrition Examination Survey; SII, systemic immunity‐inflammation index.

For this investigation, we utilized data spanning a 14‐year period (2005–2018) from the National Health and Nutrition Examination Survey (NHANES), which is one of the largest projects undertaken by the National Center for Health Statistics (NCHS). It employs intricate, multiphase, probabilistic sampling methods to gather extensive data on the dietary habits and overall health of Americans. Prior to data collection, the National Center for Health Statistics Ethics Review Committee approved all study methods, and informed consent was obtained from each participant. From 2005 to 2018, a total of 70,190 people participated in the survey. First, we excluded < 20 years old (*n* = 30,441). Second, participants without complete data on the SII (*n* = 3590) and fatigue (*n* = 3260) were also excluded. The following groups without complete data were also excluded: (1) Education level (*n* = 23); (2) Marital status (*n* = 13); (3) BMI (*n* = 303); (4) Sleep disorders (*n* = 11); (5) Vigorous activity status (*n* = 6); (6) Drinking (*n* = 32); (7) Smoking (*n* = 17); (8) Hypertension (*n* = 3). After screening, 32,491 patients in total were added to the study: 14,846 in the group for normal, 14,581 in the group for tiredness, 1520 in the group for cancer, and 1544 in the group for CRF (Figure 1).

### 2.2 Study Variables

#### 2.2.1 SII Assessment

According to previous studies [40, 41], SII is a commonly used blood inflammatory marker in the clinic. Clinical data (platelet, neutrophil, and lymphocyte counts) were obtained from routine blood tests performed at baseline for all participants. SII is calculated by multiplying the platelet count by the ratio of the neutrophil count to the lymphocyte count. Lymphocyte, neutrophil, and platelet counts were obtained by performing a complete blood count on blood specimens with a Beckman Coulter automated blood analyzer in an MEC and were expressed as × 10^3^ cells/μL.

#### 2.2.2 CRF Assessment

Using data from NHANES, the Patient Health Questionnaire‐9 (PHQ‐9) is a nine‐item instrument designed to assess depressive symptoms, based on the criteria outlined in the Diagnostic and Statistical Manual of Mental Disorders, Fourth Edition (DSM‐IV). It has been validated for use in the general population. One of the items in the PHQ‐9, specifically DPQ040, is used to ask the following questions [42]: “Over the last 2 weeks, how often have you been bothered by the following problems: feeling tired or having little energy?” Fatigue is separated between “no fatigue” (a reaction that is not exhausted at all) and “fatigue” (a response that is fatigued for many days, more than half the days, or practically every day). Of these, “several days” were classified as mild fatigue, “more than half the days” were defined as moderate fatigue, and “nearly every day” was characterized as severe fatigue. Although NHANES does not include CRF assessments, it commonly uses standalone questions to assess patient‐reported fatigue [43, 44]. Evaluate cancer outcomes using the items: “Have you ever been told by a doctor or other health professional that you have cancer or any type of malignancy?” The answer “yes” is considered to have cancer, and the answer “no” is considered not to have cancer.

The normal group was defined as individuals with no fatigue and no cancer. The fatigue group consisted of individuals experiencing fatigue without cancer. The fatigue group comprised participants who self‐reported no history of cancer but reported experiencing fatigue. Lastly, the CRF group was defined as individuals with both fatigue and cancer.

#### 2.2.3 Assessment of Covariate

Based on previous studies [45, 46, 47], we included covariates in the analysis to take into account other factors confounding the results. We included the following covariates based on NHANES interview and inspection, laboratory and questionnaire data: gender, age, race, education level, marital status, PIR (Poverty income ratio), BMI, sleep disorders, vigorous activity status, drinking, smoking, CVD (cardiovascular disease), hypertension, and diabetes.

Physician notifications of participants' heart attacks, congestive heart failure, or coronary heart disease served as the basis for the diagnosis of CVD. A systolic blood pressure of 140 mmHg or higher, a diastolic blood pressure of 90 mmHg or higher, or the use of blood pressure medication were considered indicators of hypertension. Participants were also considered to have hypertension if they answered “yes” to questions regarding hypertension on the self‐reported questionnaire. Participants were determined to have diabetes if they answered “yes” to whether they had diabetes or were taking insulin, or if they had fasting blood glucose (≥ 126 mg/dL) or glycosylated hemoglobin (≥ 6.5%). Participants were assessed for sleep disorders using the question from SLQ050: “Have you ever told a doctor or other health professional that you have a sleep disorder?” Individuals who replied “yes” to this question were characterized as having a sleep disorder and were submitted to additional testing. According to the combined PAD200 and PAD605 entries, at least 10 min of vigorous activity in the past 30 days is classified as vigorous activity. By answering the questions, participants were determined to be smokers (SMQ020) and drinkers (ALQ110, ALQ111).

### 2.3 Statistical Analysis

The NHANES data were weighted and analyzed, with participants divided into four groups based on their fatigue status and cancer diagnosis. The normal group served as the reference group, while the remaining three groups—those with fatigue without cancer, those who were cancer‐free but fatigued, and those with both fatigue and cancer—were analyzed separately. Continuous variables are expressed as mean (95% CI), and categorical variables are expressed as percentage (95% CI). A binary logistic regression model was used to calculate 95% confidence intervals (CI) and corrected odds ratios (ORs) for the factors associated with the outcome variables, respectively. The SII was divided by 1000 to magnify the effect value by a factor of 1000 [48]. In this study, three types of adjustment models were established. In the Crude Model, no covariates were shifted. In Model 1, adjustments were made for gender, age, and race. In Model 2, further adjustments included education level, marital status, PIR, BMI, sleep disorders, vigorous activity status, drinking, smoking, CVD, hypertension, and diabetes. In addition, SII/1000 was categorized into quartiles (Q1 through Q4), and a trend test was performed with Q1 serving as the reference group.

Three nodes were placed at the 10th, 50th, and 90th percentiles of SII levels, and restricted cubic splines (RCS) were used to evaluate the dose–response associations between SII and the outcomes of normal, fatigue, cancer, and CRF across each of the three models. If *p* for overall < 0.05, this indicates a significant correlation between the dependent and independent variables. If *p* for nonlinear > 0.05, it suggests that the relationship is linear. Conversely, if *p* for nonlinear < 0.05, the relationship is considered nonlinear. Additionally, a threshold effect analysis was performed to assess both the linear and nonlinear relationships between SII and the three groups, with corresponding thresholds calculated for each group. SII Inflection point = Inflection point × 1000 cells/μL. In the fully adjusted Model 2, stratified analysis was employed to explore intergroup differences among various populations. Additionally, fatigue levels were categorized into mild, moderate, and severe for subgroup analysis.

All statistical analyses were performed using SPSS (version 27.0) and R (version 4.4.0). Statistical significance was determined by *p* < 0.05.

## 3 Results

### 3.1 Baseline Characteristics

A total of 32,491 eligible individuals were selected from NHANES between 2005 and 2018, including 14,846 in the normal group, 14,581 in the fatigue group, 1520 in the cancer group, and 1544 in the CRF group, according to the screening criteria depicted in Figure 1. The weighted estimates of the baseline characteristics of the research population are shown in Table 1. Significant variations in baseline features were observed in the fatigue, cancer, and CRF groups compared to the normal group. The fatigue group is more likely to exhibit a lower PIR, higher BMI, and increased alcohol consumption. The cancer group was more likely to have a lower PIR and engage in less vigorous physical activity. The CRF group was more likely to have a higher BMI, non‐married marital status, less vigorous activity status, and a higher alcohol consumption rate.

**TABLE 1:** The research population's weighted basic features.

Characteristics	Normal	Fatigue		Cancer		CRF	
	*n* = 14,846	*n* = 14,581	*p*	*n* = 1520	*p*	*n* = 1544	*p*
SII (10^3^cells/uL) (mean)	524.95 (517.33, 532.56)	556.20 (548.25, 564.14)	**< 0.001**	580.81 (557.26, 604.35)	**< 0.001**	592.48 (571.64, 613.32)	**< 0.001**
Gender (%)			0.473		0.411		0.322
Female	49.13 (48.04, 50.21)	48.54 (47.40, 49.68)		50.40 (47.41, 53.39)		47.42 (44.11, 50.75)	
Male	50.87 (49.79, 51.96)	51.46 (50.32, 52.60)		49.60 (46.61, 52.59)		52.58 (49.25, 55.89)	
Age (years) (%)			0.138		0.852		0.655
20–39	32.57 (31.27, 33.89)	34.07 (32.81, 35.35)		33.12 (30.36, 35.99)		33.34 (30.22, 36.62)	
40–59	32.44 (31.11, 33.80)	31.28 (30.06, 32.54)		32.81 (29.56, 36.24)		33.18 (30.14, 36.35)	
≥ 60	34.99 (33.84, 36.16)	34.65 (33.52, 35.79)		34.07 (30.69, 37.62)		33.48 (30.55, 36.54)	
Race (%)			0.087		0.548		0.057
Mexican American	15.71 (14.98, 16.47)	16.15 (15.35, 16.98)		16.87 (14.57, 19.45)		16.49 (14.55, 18.64)	
Other Hispanic	9.20 (8.59, 9.85)	9.89 (9.23, 10.60)		9.02 (7.20, 11.25)		8.73 (7.32, 10.39)	
Non‐Hispanic white	43.94 (43.01, 44.88)	41.98 (40.92, 43.05)		45.35 (42.36, 48.39)		46.89 (43.71, 50.10)	
Non‐Hispanic black	20.93 (20.03, 21.86)	20.89 (19.89, 21.92)		19.99 (17.52, 22.70)		20.17 (17.70, 22.90)	
Other race	10.22 (9.54, 10.94)	11.09 (10.39, 11.82)		8.77 (7.00, 10.93)		7.71 (6.20, 9.54)	
Education Level (%)			0.576		0.059		0.253
Less than high school	24.30 (23.25, 25.38)	24.92 (24.10, 25.75)		21.67 (18.97, 24.62)		23.98 (21.02, 27.22)	
High school diploma	22.55 (21.70, 23.44)	22.43 (21.58, 23.30)		25.73 (22.90, 28.77)		24.91 (21.73, 28.39)	
More than high school	53.15 (51.99, 54.30)	52.65 (51.59, 53.72)		52.61 (48.94, 56.25)		51.11 (48.15, 54.06)	
Marital Status (%)			0.128		0.418		**0.006**
Married	52.87 (51.62, 54.12)	52.29 (51.10, 53.49)		53.33 (49.80, 56.82)		53.72 (50.70, 56.71)	
Living with partner	8.03 (7.37, 8.74)	9.07 (8.40, 9.79)		9.27 (7.24, 11.79)		9.35 (7.48, 11.62)	
Divorced/widowed/separated	21.33 (20.40, 22.29)	20.95 (19.99, 21.94)		21.53 (18.45, 24.96)		23.44 (20.67, 26.46)	
Never married	17.77 (16.74, 18.84)	17.69 (16.76, 18.65)		15.88 (13.79, 18.22)		13.49 (11.28, 16.07)	
PIR (%)			**< 0.001**		**< 0.001**		0.264
< 2	29.78 (28.32, 31.28)	34.59 (32.76, 36.46)		20.10 (17.64, 22.81)		31.65 (28.17, 35.35)	
≥ 2	70.22 (68.72, 71.68)	65.41 (63.54, 67.24)		79.90 (77.19, 82.36)		68.35 (64.65, 71.83)	
BMI (kg/m^2^) (%)			**< 0.001**		0.531		**< 0.001**
< 25	30.65 (29.31, 32.03)	28.61 (27.45, 29.78)		28.97 (26.17, 31.95)		26.01 (23.34, 28.87)	
25–29.99	35.55 (34.48, 36.63)	30.14 (29.18, 31.12)		37.18 (33.63, 40.87)		32.82 (30.11, 35.66)	
≥ 30	33.80 (32.46, 35.17)	41.25 (40.03, 42.49)		33.85 (30.71, 37.13)		41.17 (38.39, 44.01)	
Sleep disorders (%)			**< 0.001**		**< 0.001**		**< 0.001**
No	83.05 (82.08, 83.99)	64.14 (62.83, 65.42)		74.85 (71.63, 77.81)		49.25 (46.46, 52.05)	
Yes	16.95 (16.01, 17.92)	35.86 (34.58, 37.17)		25.15 (22.19, 28.37)		50.75 (47.95, 53.54)	
Vigorous activity status (%)			0.134		**< 0.001**		**< 0.001**
No	74.61 (73.34, 75.85)	75.77 (74.63, 76.86)		81.47 (78.50, 84.12)		82.61 (79.62, 85.24)	
Yes	25.39 (24.15, 26.66)	24.23 (23.14, 25.37)		18.53 (15.88, 21.50)		17.39 (14.76, 20.38)	
Drinking (%)			**< 0.001**		0.464		**0.010**
No	11.55 (10.60, 12.58)	9.86 (8.92, 10.87)		10.67 (8.66, 13.09)		9.15 (7.50, 11.11)	
Yes	88.45 (87.42, 89.40)	90.14 (89.13, 91.08)		89.33 (86.91, 91.34)		90.85 (88.89, 92.50)	
Smoking (%)			**< 0.001**		**< 0.001**		**< 0.001**
No	57.82 (56.47, 59.15)	53.73 (52.25, 55.20)		50.28 (47.04, 53.53)		43.14 (39.73, 46.61)	
Yes	42.18 (40.85, 43.53)	46.27 (44.80, 47.75)		49.72 (46.47, 52.96)		56.86 (53.39, 60.27)	
CVD (%)			**0.005**		**< 0.001**		**< 0.001**
No	95.32 (94.73, 95.85)	94.32 (93.76, 94.83)		88.52 (86.32, 90.41)		83.98 (81.52, 86.16)	
Yes	4.68 (4.15, 5.27)	5.68 (5.17, 6.24)		11.48 (9.59, 13.68)		16.02 (13.84, 18.48)	
Hypertension (%)			**0.003**		**< 0.001**		**< 0.001**
No	65.42 (64.21, 66.60)	63.35 (62.03, 64.66)		43.36 (39.62, 47.17)		40.11 (37.03, 43.27)	
Yes	34.58 (33.40, 35.79)	36.65 (35.34, 37.97)		56.64 (52.83, 60.38)		59.89 (56.73, 62.97)	
Diabetes (%)			**< 0.001**		**< 0.001**		**< 0.001**
No	89.13 (88.33, 89.88)	86.87 (86.10, 87.61)		81.19 (78.57, 83.55)		77.22 (74.99, 79.31)	
Yes	10.87 (10.12, 11.67)	13.13 (12.39, 13.90)		18.81 (16.45, 21.43)		22.78 (20.69, 25.01)	

*Note:* Bold indicates *p* < 0.05.

Abbreviations: BMI, body mass index; CI, confidence interval; CRF, cancer‐related fatigue; CVD, cardiovascular disease; OR, odds ratio; PIR, Poverty income ratio; SII, systemic immunity‐inflammation index.

### 3.2 Association Between SII and CRF

The results showed that SII was positively correlated with the Fatigue group, Cancer group, and CRF group. Table 2 showed the results in a completely confound‐adjusted model (Model 2), in which increased SII was strongly correlated with a 43% increase in the incidence of Fatigue, a 67% increase in cancer, and a 93% increase in CRF. The quartile analysis and *p* for trend results further indicated that, compared to the first quartile (Q1), the ORs for the fourth quartile (Q4) were 1.30 (1.22, 1.39), 1.60 (1.38, 1.87), and 1.82 (1.56, 2.13), with *p*‐values for all three groups being less than 0.001. Additionally, the *p* for trend was below 0.001. As SII increased, the incidence of fatigue, cancer, and CRF also rose significantly, with the most pronounced increase observed in the CRF group.

**TABLE 2:** Relationship between SII and fatigue, cancer, and CRF in model II.

Outcome	Fatigue	Cancer	CRF
	OR (95% CI)	OR (95% CI)	OR (95% CI)
SII/1000	1.43 (1.33, 1.55)	1.67 (1.43, 1.95)	1.93 (1.66, 2.25)
SII/1000 quartile			
Q1	Reference		
Q2	1.05 (0.98, 1.12)	1.12 (0.95, 1.32)	1.15 (0.97, 1.36)
Q3	1.12 (1.05, 1.20)	1.24 (1.05, 1.45)	1.13 (0.95, 1.33)
Q4	1.30 (1.22, 1.39)	1.60 (1.38, 1.87)	1.82 (1.56, 2.13)
*p* for trend	1.09 (1.06, 1.11)	1.17 (1.11, 1.23)	1.21 (1.15, 1.27)

*Note:* Non‐adjusted model adjust for: none; adjust I model adjust for: gender, age, race; adjust II model adjust for: gender, age, race, education level, marital status, PIR, BMI, sleep disorders, vigorous activity status, drinking, smoking, CVD, hypertension, and diabetes.

Abbreviations: CI, confidence interval; OR, odds ratio.

**FIGURE 2:**
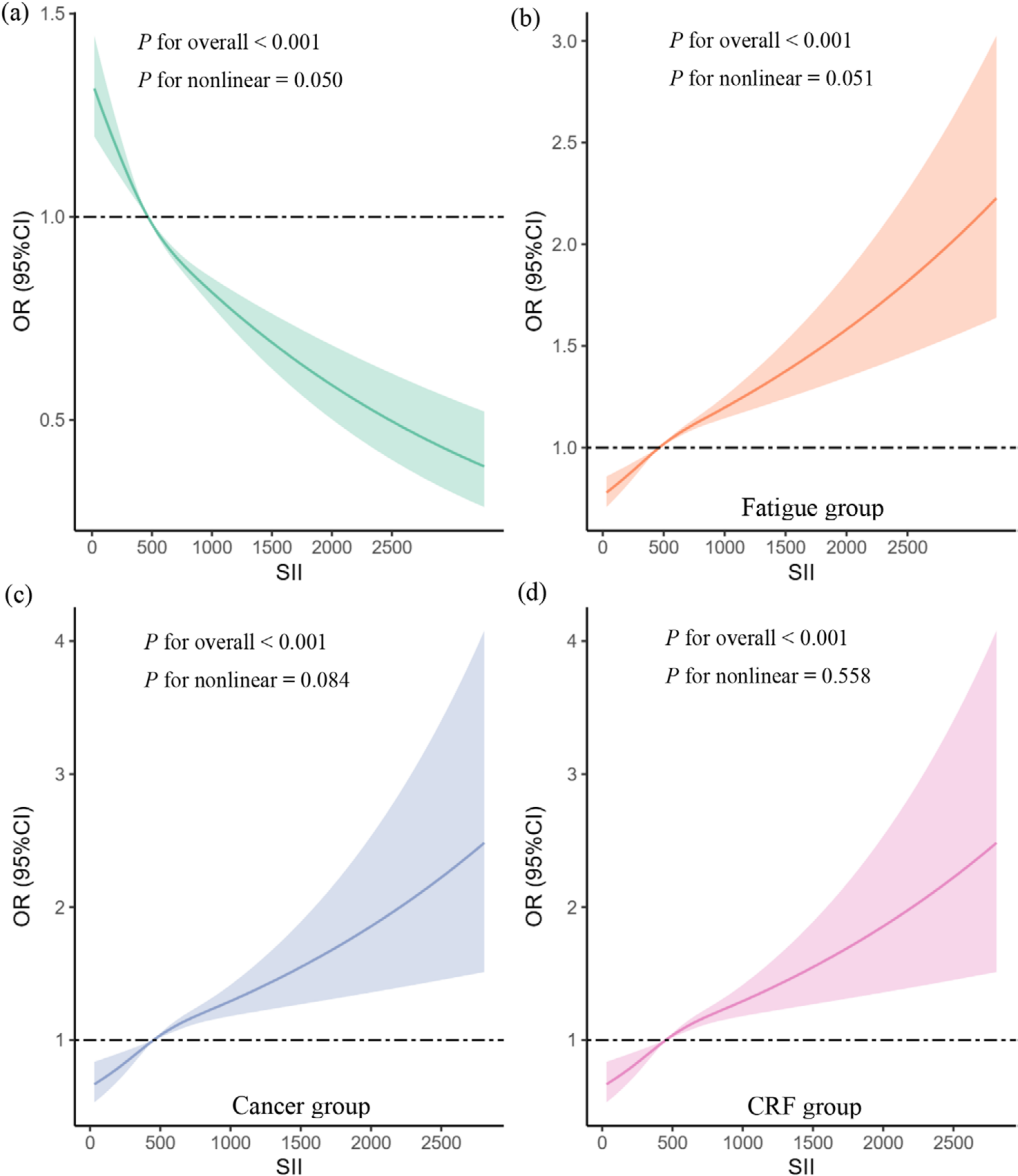
SII dose–response analysis and (a) normal group, (b) fatigue group, (c) cancer group, (d) CRF group prevalence (RCS). (a), (b), (c), (d): whole modified model; (a) the normal group was compared with the other three groups. CI, confidence interval; CRF, cancer‐related fatigue; OR, odds ratio; RCS, restricted cubic splines; SII, systemic immunity‐inflammation index.

Figure 2 illustrates the results of the restricted cubic spline (RCS) analysis, depicting the dose–response relationship. Under the fully adjusted model, the incidence of SII is in a linear relationship with Fatigue (Figure 2b), cancer (Figure 2c) and CRF (Figure 2d) (*p* for nonlinearity = 0.051, 0.084, 0.558, respectively). This suggests that as SII increases, the incidence of fatigue, cancer, and CRF also shows a linear increase. The threshold effects of the SII on the fatigue, cancer, CRF groups. In the fully adjusted model, the threshold values (k) for each of these groups were identified as 464.78 × 10^3^ cells/μL, 448.97 × 10^3^ cells/μL, and 454.65 × 10^3^ cells/μL, respectively (Table 3). These values can serve as benchmarks for determining normal SII levels in future studies.

**TABLE 3:** Relationship between SII/1000 and fatigue, cancer, and CRF (analysis of the threshold effect).

Outcome	Fatigue		Cancer		CRF	
	OR (95% CI)	*P*	OR (95% CI)	*P*	OR (95% CI)	*P*
Model 1						
Linear impact	1.43 (1.33, 1.55)	**< 0.001**	1.67 (1.43, 1.95)	**< 0.001**	1.93 (1.66, 2.25)	**< 0.001**
Model 2						
Inflection point (k)*	0.46478		0.44897		0.45465	
SII/1000 < k	1.68 (1.29, 2.20)	0.001	2.41 (1.24, 4.69)	0.010	1.82 (0.93, 3.53)	0.080
SII/1000 > K	1.37 (1.24, 1.52)	< 0.001	1.56 (1.28, 1.90)	< 0.001	1.95 (1.61, 2.36)	< 0.001
Log likelihood ratio	0.219		0.265		0.855	

*Note:* This model included adjustments for all covariates. Bold indicates *p* < 0.05.

Abbreviations: CI, confidence interval; CRF, cancer‐related fatigue; OR, odds ratio; SII, systemic immunity‐inflammation index.

### 3.3 Stratified and Subgroup Analysis

**FIGURE 3:**
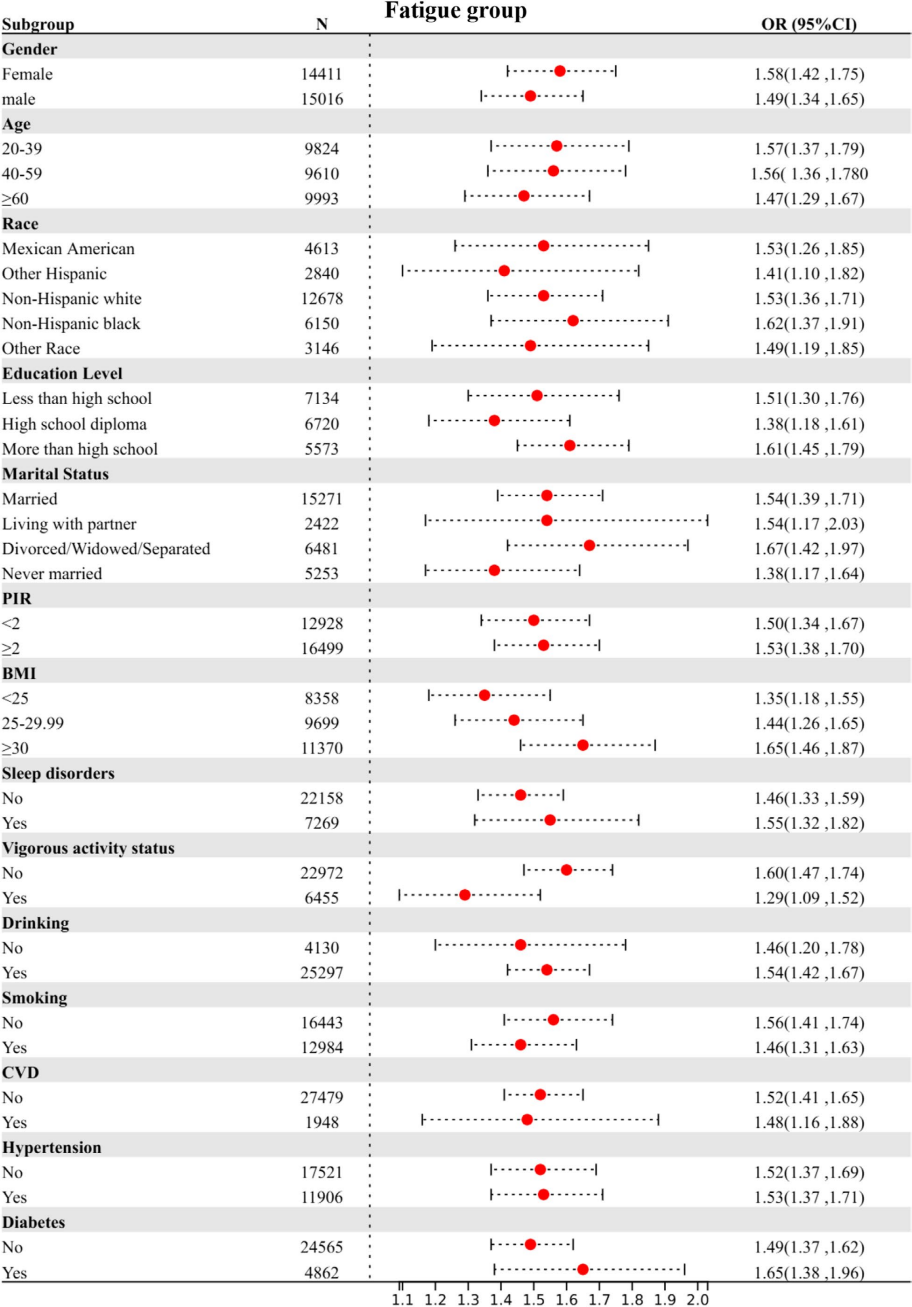
Stratified relationships between SII and fatigue prevalence in US populations. CI, confidence interval; CVD, cardiovascular disease; OR, odds ratio; SII, systemic immunity‐inflammation index.

**FIGURE 4:**
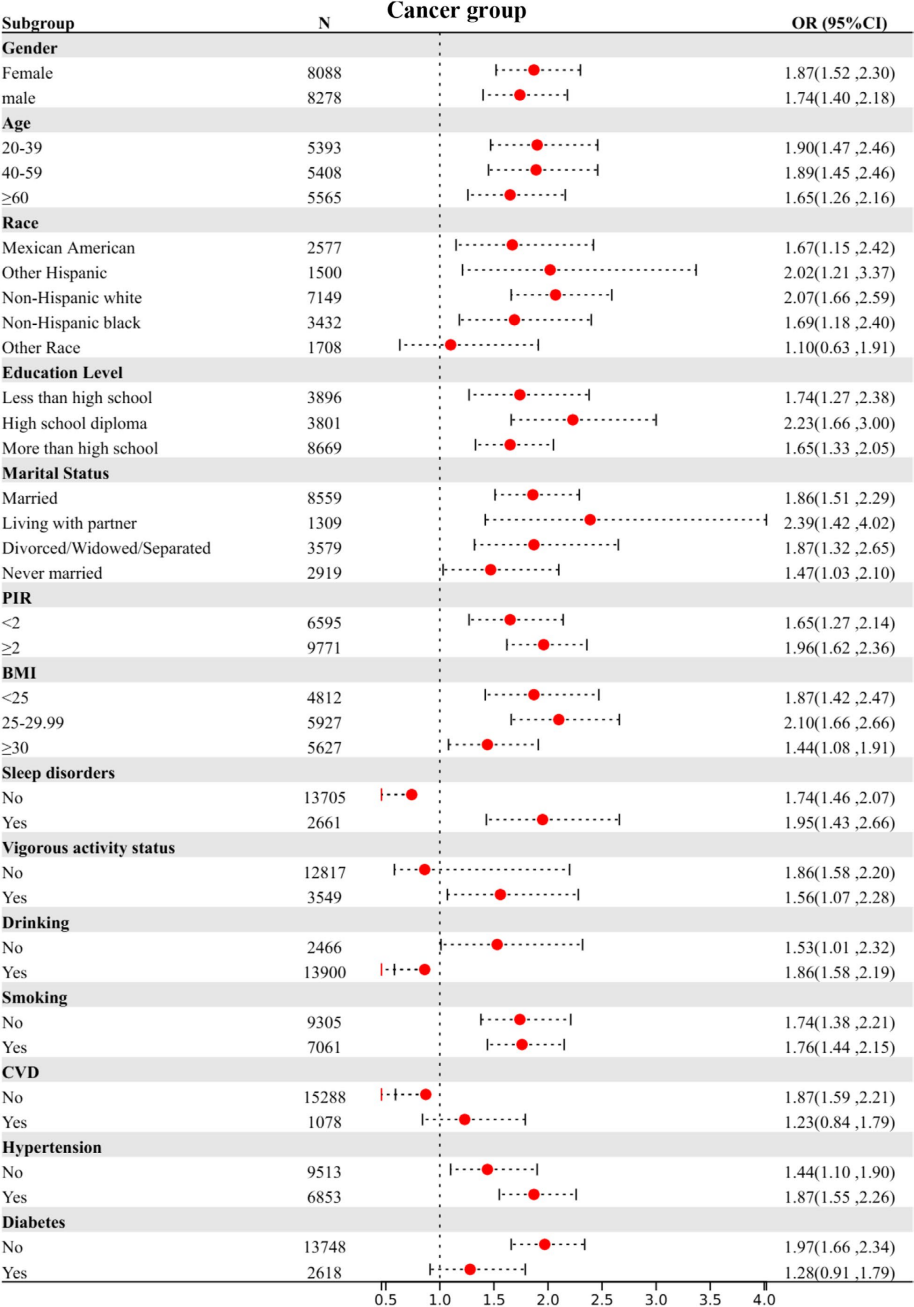
Stratified relationships between SII and cancer prevalence in US populations. BMI, body mass index; CI, confidence interval; CVD, cardiovascular disease; OR, odds ratio; PIR, poverty income ratio; SII, systemic immunity‐inflammation index.

**FIGURE 5:**
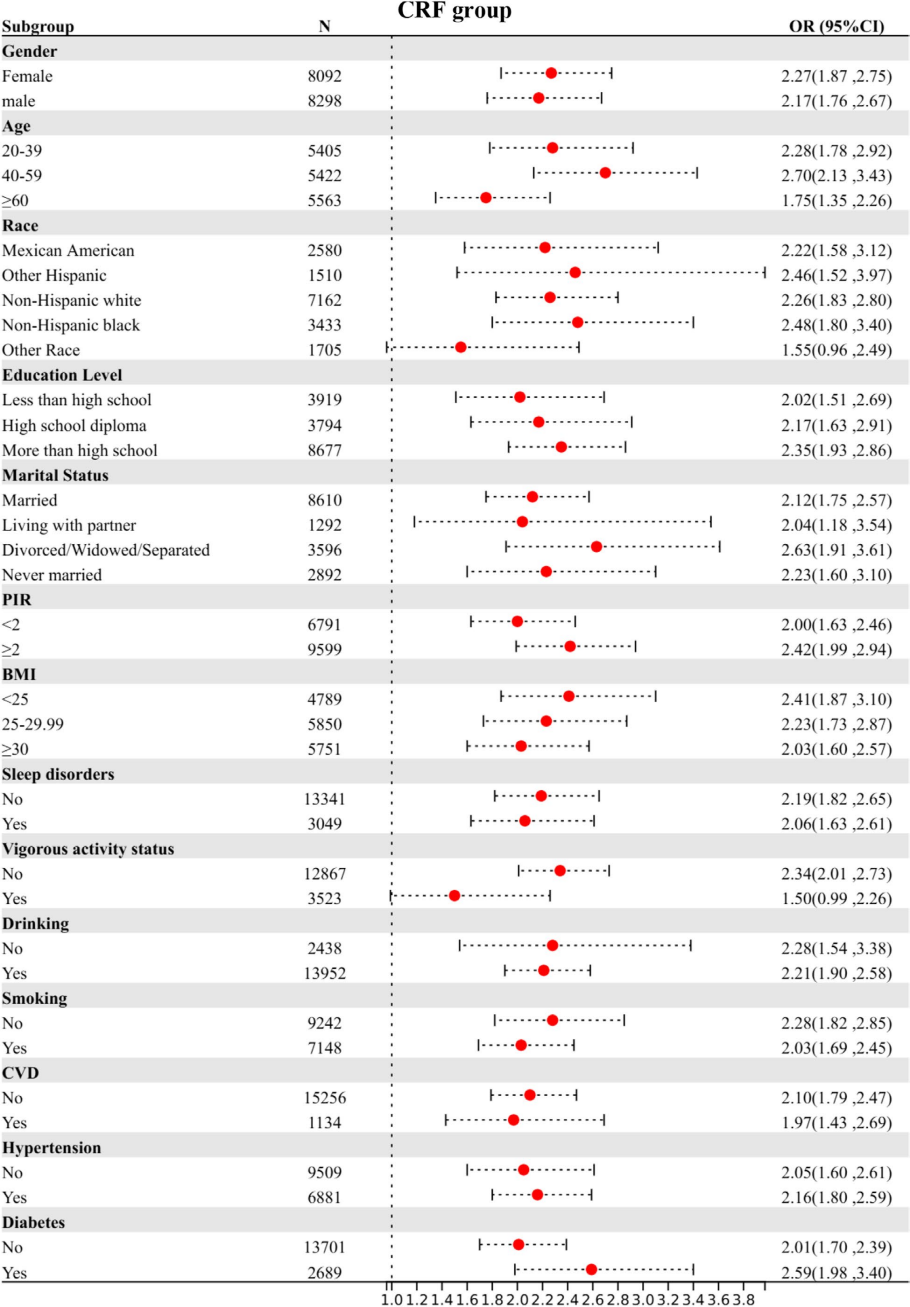
Stratified relationships between SII and CRF prevalence in US populations. BMI, body mass index; CI, confidence interval; CRF, cancer‐related fatigue; CVD, cardiovascular disease; OR, odds ratio; PIR, poverty income ratio; SII, systemic immunity‐inflammation index.

Subgroup stratification is based on the following variables: gender, age, race, education level, marital status, PIR (Poverty income ratio), BMI, sleep disorders, vigorous activity status, drinking, smoking, CVD (cardiovascular disease), hypertension, and diabetes. Figures 3, 4, 5 illustrate the results of three subgroup analyses within the population. Taking the fatigue group as an example, females aged 20–39, non‐Hispanic Black individuals, those with higher education, a PIR ≥ 2, those living with a partner, individuals with a BMI > 30, those with sleep disorders, and those who engage in no vigorous activity, consume alcohol, or have comorbid hypertension and diabetes are more likely to experience fatigue compared to other groups. In the fatigue group (*p* for interaction = 0.024) and the CRF group (*p* for interaction = 0.045), vigorous activity was found to significantly interact with SII. Similarly, in the cancer group, both CVD and diabetes showed significant interactions with SII, with *p* for interaction of 0.045 and 0.025, respectively (Table 4). The analyses revealed nearly significant differences among each stratified population, indicating that the main analysis results can be regarded as stable. The subgroup analysis results between the fatigue group and the CRF group are shown in Table 5. The results indicated that as SII increased, the degree of fatigue also progressively increased. Notably, the incidence of severe fatigue was highest in the CRF population, showing an increase of 171% (*p* < 0.001).

**TABLE 4:** Stratified relationships and *p*‐interaction between SII, fatigue, cancer, and CRF groups.

Subgroup	Fatigue		Cancer		CRF	
	OR (95% CI)	*p*‐interaction	OR (95% CI)	*p*‐interaction	OR (95% CI)	*p*‐interaction
Gender (%)		0.437		0.663		0.752
Female	1.58 (1.42, 1.75)		1.87 (1.52, 2.30)		2.27 (1.87, 2.75)	
Male	1.49 (1.34, 1.65)		1.74 (1.40, 2.18)		2.17 (1.76, 2.67)	
Age (years) (%)		0.733		0.712		0.050
20–39	1.57 (1.37, 1.79)		1.90 (1.47, 2.46)		2.28 (1.78, 2.92)	
40–59	1.56 (1.36, 1.780		1.89 (1.45, 2.46)		2.70 (2.13, 3.43)	
≥ 60	1.47 (1.29, 1.67)		1.65 (1.26, 2.16)		1.75 (1.35, 2.26)	
Race (%)		0.925		0.247		0.549
Mexican American	1.53 (1.26, 1.85)		1.67 (1.15, 2.42)		2.22 (1.58, 3.12)	
Other Hispanic	1.41 (1.10, 1.82)		2.02 (1.21, 3.37)		2.46 (1.52, 3.97)	
Non‐Hispanic white	1.53 (1.36, 1.71)		2.07 (1.66, 2.59)		2.26 (1.83, 2.80)	
Non‐Hispanic black	1.62 (1.37, 1.91)		1.69 (1.18, 2.40)		2.48 (1.80, 3.40)	
Other race	1.49 (1.19, 1.85)		1.10 (0.63, 1.91)		1.55 (0.96, 2.49)	
Education level (%)		0.254		0.264		0.690
Less than high school	1.51 (1.30, 1.76)		1.74 (1.27, 2.38)		2.02 (1.51, 2.69)	
High school diploma	1.38 (1.18, 1.61)		2.23 (1.66, 3.00)		2.17 (1.63, 2.91)	
More than high school	1.61 (1.45, 1.79)		1.65 (1.33, 2.05)		2.35 (1.93, 2.86)	
Marital status (%)		0.487		0.468		0.715
Married	1.54 (1.39, 1.71)		1.86 (1.51, 2.29)		2.12 (1.75, 2.57)	
Living with partner	1.54 (1.17, 2.03)		2.39 (1.42, 4.02)		2.04 (1.18, 3.54)	
Divorced/widowed/separated	1.67 (1.42, 1.97)		1.87 (1.32, 2.65)		2.63 (1.91, 3.61)	
Never married	1.38 (1.17, 1.64)		1.47 (1.03, 2.10)		2.23 (1.60, 3.10)	
PIR (%)		0.785		0.301		0.191
< 2	1.50 (1.34, 1.67)		1.65 (1.27, 2.14)		2.00 (1.63, 2.46)	
≥ 2	1.53 (1.38, 1.70)		1.96 (1.62, 2.36)		2.42 (1.99, 2.94)	
BMI (kg/m^2^) (%)		0.090		0.125		0.615
< 25	1.35 (1.18, 1.55)		1.87 (1.42, 2.47)		2.41 (1.87, 3.10)	
25–29.99	1.44 (1.26, 1.65)		2.10 (1.66, 2.66)		2.23 (1.73, 2.87)	
≥ 30	1.65 (1.46, 1.87)		1.44 (1.08, 1.91)		2.03 (1.60, 2.57)	
Sleep disorders (%)		0.502		0.521		0.686
No	1.46 (1.33, 1.59)		1.74 (1.46, 2.07)		2.19 (1.82, 2.65)	
Yes	1.55 (1.32, 1.82)		1.95 (1.43, 2.66)		2.06 (1.63, 2.61)	
Vigorous activity status (%)		**0.024**		0.402		**0.045**
No	1.60 (1.47, 1.74)		1.86 (1.58, 2.20)		2.34 (2.01, 2.73)	
Yes	1.29 (1.09, 1.52)		1.56 (1.07, 2.28)		1.50 (0.99, 2.26)	
Drinking (%)		0.618		0.393		0.893
No	1.46 (1.20, 1.78)		1.53 (1.01, 2.32)		2.28 (1.54, 3.38)	
Yes	1.54 (1.42, 1.67)		1.86 (1.58, 2.19)		2.21 (1.90, 2.58)	
Smoking (%)		0.392		0.962		0.441
No	1.56 (1.41, 1.74)		1.74 (1.38, 2.21)		2.28 (1.82, 2.85)	
Yes	1.46 (1.31, 1.63)		1.76 (1.44, 2.15)		2.03 (1.69, 2.45)	
CVD (%)		0.818		**0.045**		0.707
No	1.52 (1.41, 1.65)		1.87 (1.59, 2.21)		2.10 (1.79, 2.47)	
Yes	1.48 (1.16, 1.88)		1.23 (0.84, 1.79)		1.97 (1.43, 2.69)	
Hypertension (%)		0.940		0.125		0.722
No	1.52 (1.37, 1.69)		1.44 (1.10, 1.90)		2.05 (1.60, 2.61)	
Yes	1.53 (1.37, 1.71)		1.87 (1.55, 2.26)		2.16 (1.80, 2.59)	
Diabetes (%)		0.327		**0.025**		0.122
No	1.49 (1.37, 1.62)		1.97 (1.66, 2.34)		2.01 (1.70, 2.39)	
Yes	1.65 (1.38, 1.96)		1.28 (0.91, 1.79)		2.59 (1.98, 3.40)	

*Note:* This model included adjustments for all covariates. Bold indicates *p* < 0.05.

Abbreviations: BMI, body mass index; CI, confidence interval; CRF, cancer‐related fatigue; CVD, cardiovascular disease; OR, odds ratio; PIR, Poverty income ratio; SII, systemic immunity‐inflammation index.

**TABLE 5:** Relationship between SII and fatigue level.

Fatigue level	Fatigue		CRF	
	OR (95% CI)	*p*	OR (95% CI)	*p*
No	Reference			
Mild	1.37 (1.26, 1.50)	**< 0.001**	1.64 (1.35, 2.00)	< 0.001
Moderate	1.41 (1.22, 1.61)	**< 0.001**	1.91 (1.39, 2.61)	**< 0.001**
Severe	1.74 (1.53, 1.97)	**< 0.001**	2.71 (2.12, 3.47)	**< 0.001**

*Note:* Non‐adjusted model adjust for: None; adjust I model adjust for: Gender, age, race; Adjust II model adjust for: Gender, age, race, education level, marital status, PIR, BMI, sleep disorders, vigorous activity status, drinking, smoking, CVD, hypertension, and diabet. Bold indicates *p* < 0.05.

Abbreviations: BMI, body mass index; CI, confidence interval; CRF, cancer‐related fatigue; OR, odds ratio; PIR, poverty income ratio; SII, systemic immunity‐inflammation index.

## 4 Discussion

This retrospective cross‐sectional study is the first to explore the relationship between SII and fatigue, cancer, and CRF, revealing a positive correlation among these three groups after adjusting for confounding factors. Subsequent analysis using restricted cubic splines revealed a linear increase in the incidence rates of fatigue, cancer, and CRF with higher SII levels among American populations. The quartile analysis and dose–response relationship showed that increasing SII levels were significantly associated with higher risks of fatigue, cancer, and CRF. This gradient in risk, particularly pronounced for CRF, may reflect the exacerbation of inflammatory and immunological processes at elevated SII levels, highlighting its potential utility in risk stratification. The identified thresholds were 464.78 × 10^3^ cells/μL, 448.97 × 10^3^ cells/μL, and 454.65 × 10^3^ cells/μL, respectively, indicating that higher SII levels are associated with increased frequencies of these outcomes. Additionally, detailed analyses in three subpopulations were conducted. Although various factors were considered in the fully adjusted model, we were unable to capture all the factors influencing fatigue. A substantial body of literature has shown that individuals with depression and chronic pain often exhibit significant fatigue symptoms [49, 50], which are closely associated with the physiological and psychological mechanisms of their conditions. It may also be worth considering combining multiple disease groups to assess their synergistic effects on fatigue, thereby developing more comprehensive intervention strategies. Therefore, maintaining appropriate SII levels may be a promising strategy for preventing fatigue, cancer, and CRF.

The mechanism of CRF is complex, but two primary theoretical frameworks are commonly used to explain its mechanisms: central (neural) and peripheral (muscle) mechanisms [51]. The central mechanism involves disruptions such as circadian rhythm disturbances [52], changes in cytokine activity [53], and alterations in serotonin levels [54]. These disruptions activate peripheral pro‐inflammatory cytokine networks and impair physiological barrier integrity, leading to central nervous system inflammation, interference with hypothalamic–pituitary–adrenal (HPA) axis function, and contributing to CRF [55]. In contrast, peripheral mechanisms focus on the role of muscle contraction characteristics and adenosine triphosphate (ATP) metabolism in the development of CRF [56, 57].

Inflammatory cytokines have garnered significant attention because they can act as targets for tumors, potentially aiding in cancer treatment [58]. A comprehensive review underscores a strong association between CRF and various inflammatory cytokines (IL‐6, IL‐8, IL‐10, IL‐1β), TNF‐α, catechol‐O‐methyltransferase (COMT), and circadian rhythm genes [59]. Although the precise mechanisms linking SII to CRF are not fully understood, partly due to the complex interactions among neutrophils, platelets, and lymphocytes in the SII, the neutrophil count is a key indicator of the innate immune system's activity, and its variation reflects the activation state of the immune response [60]. An increase in the neutrophil count is associated with the upregulation of pro‐inflammatory cytokines, which in turn promote tumor proliferation, invasion, and metastasis. Furthermore, neutrophils release extracellular traps that facilitate distant metastasis [61]. Higher neutrophil counts are linked not only to increased levels of fatigue but also to a greater intensity of emotional fatigue, particularly in individuals with CRF [62].

Platelets, on the other hand, release growth factors that protect tumor cells from immune surveillance, thus promoting tumor growth and angiogenesis [63]. Elevated platelet counts have been shown to support tumor progression and metastatic potential through various biological mechanisms, including the secretion of growth factors and the stimulation of angiogenesis and tumor growth [64]. In contrast, lymphocytes primarily engage in the adaptive immune response, with critical regulatory functions in antitumor immunity. A reduction in peripheral lymphocyte count can impair immune effectiveness, negatively affecting cancer prognosis [65]. Lower lymphocyte counts, particularly in CD4 T cells and natural killer cells, have been correlated with increased levels of physical fatigue in cancer survivors. This pattern may reflect the link between the immune system's response to tumors or their treatment and physical fatigue, or it may indicate a pathophysiological mechanism that triggers both immune system activation and fatigue [66]. In summary, SII, as a composite index, reflects the delicate balance between the inflammatory responses of neutrophils and platelets and the antitumor immune function system of lymphocytes. This balance, to some extent, reveals the potential connection with CRF.

The stratified analysis revealed significant differences between patients with fatigue, cancer, and CRF, particularly highlighting that women are more prone to fatigue than men. This finding is supported by recent meta‐analytic evidence [67]. The analysis also identified variations in fatigue, cancer, and CRF based on factors such as age, marital status, race, PIR, BMI, sleep disorders, drinking, smoking, and comorbidities. Existing research highlights that conditions such as hypertension, diabetes, and cardiovascular diseases can amplify the production of inflammatory cytokines, potentially worsening CRF [68, 69, 70]. Furthermore, a lack of physical activity is strongly associated with higher levels of fatigue [71].

The interaction results indicate that individuals who do not engage in vigorous physical activity within the fatigue and CRF groups exhibit higher values of the SII. Regular physical activity significantly reduces fatigue by modulating the immune system, decreasing systemic inflammation, and enhancing physical endurance [72]. Furthermore, these findings suggest that while CVD and diabetes are well‐established health risk factors, individuals without cardiovascular disease may be more susceptible to the adverse effects of cancer. Thus, early identification and understanding of the factors associated with CRF are essential. This approach is crucial for developing personalized intervention strategies tailored to different cancer stages and types, with the goal of reducing patient suffering and enhancing treatment outcomes.

This study has notable strengths: First, it uses a large sample of 32,191 participants over 14 years from a nationally representative database, ensuring broad and accurate data. Second, it explores not only the relationship between SII and CRF but also examines SII's connections with fatigue and cancer itself, providing a multidimensional analytical perspective. Third, it innovatively considers SII as a potential tool for early CRF identification, emphasizing its economic, safe, and efficient attributes.

However, there are several limitations to this study: First, self‐reported questionnaires might introduce recall bias, impacting result accuracy. Second, the cross‐sectional design prevents direct inference of causal relationships between SII and CRF. Future research could involve cohort studies to further investigate whether the trend of fatigue evolves over time. Third, despite efforts to control for confounding variables, biases from unidentified factors such as pain or depression may still exist. Fourth, due to limitations in the database, fatigue status could not be assessed using comprehensive scales and was instead evaluated based on single‐item data. Measuring fatigue through individual items, rather than using a comprehensive scale, can lead to measurement errors. This limitation makes it challenging to accurately differentiate between types of fatigue, such as mental and physical fatigue. Despite these drawbacks, their simplicity and convenience make them popular tools for screening large populations. Future research should address these limitations by incorporating validated multidimensional fatigue scales, such as the Fatigue Assessment Scale (FAS), to better elucidate the complex relationship between SII and CRF.

## 5 Conclusion

The study results indicate a significant linear positive correlation between SII and the incidence of CRF, which is also observed in both fatigue and cancer populations. Higher SII is specifically associated with increased smoking rates, a higher prevalence of sleep disorders, and a greater likelihood of developing cardiovascular disease, hypertension, and a history of diabetes. It is noteworthy that the expression of this relationship varies across different populations. Generally, higher values of SII are associated with greater severity of fatigue, with this trend being particularly pronounced in CRF.

## Author Contributions

**Li Sun:** data curation, methodology, investigation, software, validation, writing – review and editing, writing – original draft. **Yanling Wu:** methodology, software, visualization, writing – review and editing. **Lydia Idowu Akinyemi:** methodology, conceptualization, visualization. **Zhiqiu Cao:** methodology, validation, visualization, software. **Zhanhong Fan:** conceptualization, data curation, supervision, investigation. **Huahua Liu:** project administration, writing – review and editing, grammar modification, resources. **Ziyi Yang:** methodology, validation, project administration, visualization. **Leilei Zhang:** writing – review and editing, grammar modification. **Feng Zhang:** supervision, resources, writing – review and editing, software, funding acquisition.

## Conflicts of Interest

The authors declare no conflicts of interest.

## Data Availability

The data supporting the findings of this study are available from the corresponding author upon request.
